# Supplementary material for: Revisiting the Woolly wolf (Canis lupus chanco) phylogeny in Himalaya: Addressing taxonomy, spatial extent and distribution of an ancient lineage in Asia
Source: PLoS One. 2020 Apr 16;15(4):e0231621. doi: 10.1371/journal.pone.0231621 (PMC7162449; doi:10.1371/journal.pone.0231621)
Supplement: S1 Table — (DOCX) [file pone.0231621.s001.docx]

Table S1. Details of samples and Sequences used in this study

| **SN** | **Accession No** |  | **Location** | **Citations** | **Specific name** |
| --- | --- | --- | --- | --- | --- |
| Seq1 | AY289973.1 | *Canis indica* | India | Aggarwal et al., 2007 |  |
| Seq2 | AY289974.1 | *Canis indica* | India | Aggarwal et al., 2007 |  |
| Seq3 | AY289975.1 | *Canis indica* | India | Aggarwal et al., 2007 |  |
| Seq4 | AY289976.1 | *Canis indica* | India | Aggarwal et al., 2007 |  |
| Seq5 | AY333744.1 | *Canis lupus pallipes* | India | Sharma et al., 2003 |  |
| Seq6 | AY333743.1 | *Canis lupus pallipes* | India | Aggarwal et al., 2007 |  |
| Seq7 | NC_010340.2 | *Canis lupus chanco* | Mongolia | Meng and Zhang, 2008* |  |
| Seq8 | 81 | *Canis lupus chanco* | India | Present Study |  |
| Seq9 | 73 | *Canis lupus chanco* | India | Present Study |  |
| Seq10 | 39 | *Canis lupus chanco* | India | Present Study |  |
| Seq11 | 34 | *Canis lupus chanco* | India | Present Study |  |
| Seq12 | 89 | *Canis lupus chanco* | India | Present Study |  |
| Seq13 | 36 | *Canis lupus chanco* | India | Present Study |  |
| Seq14 | 41 | *Canis lupus chanco* | India | Present Study |  |
| Seq15 | 71 | *Canis lupus chanco* | India | Present Study |  |
| Seq16 | 46 | *Canis lupus chanco* | India | Present Study |  |
| Seq17 | 53 | *Canis lupus chanco* | India | Present Study |  |
| Seq18 | 50 | *Canis lupus chanco* | India | Present Study |  |
| Seq19 | 40 | *Canis lupus chanco* | India | Present Study |  |
| Seq20 | 83 | *Canis lupus chanco* | India | Present Study |  |
| Seq21 | 75 | *Canis lupus chanco* | India | Present Study |  |
| Seq22 | 66 | *Canis lupus chanco* | India | Present Study |  |
| Seq23 | 63 | *Canis lupus chanco* | India | Present Study |  |
| Seq24 | 64 | *Canis lupus chanco* | India | Present Study |  |
| Seq25 | AY333738.1 | *Canis lupus chanco* | India | Sharma et al., 2003 |  |
| Seq26 | JX415344.1 | *Canis lupus chanco* | China, | Dou, 2012* |  |
| Seq27 | AY333746.1 | *Canis lupus pallipes* | India | Sharma et al., 2003 |  |
| Seq28 | AY333742.1 | *Canis lupus chanco* | India | Sharma et al., 2003 |  |
| Seq29 | AY333745.1 | *Canis lupus pallipes* | India | Sharma et al., 2003 |  |
| Seq30 | AB007374.1 | *Canis lupus pallipes* | India | Tsuda et al., 1997 |  |
| Seq31 | AB007378.2 | *Canis lupus chanco* | Mongolia | Tsuda et al., 1997 |  |
| Seq32 | AB007377.2 | *Canis lupus chanco* | Mongolia | Tsuda et al., 1997 |  |
| Seq33 | AB007379.1 | *Canis lupus chanco* | Mongolia | Tsuda et al., 1997 |  |
| Seq34 | AB007376.1 | *Canis lupus chanco* | Mongolia | Tsuda et al., 1997 |  |
| Seq35 | KC414579.1 | *Canis lupus chanco* | Mongolia | Dou and Zhang, 2012* | Mongolian wolf |
| Seq36 | KC414578.1 | *Canis lupus chanco* | Mongolia | Dou and Zhang, 2012 | Mongolian wolf |
| Seq37 | KC414577.1 | *Canis lupus chanco* | Mongolia | Dou and Zhang, 2012 | Mongolian wolf |
| Seq38 | AB007375.1 | *Canis lupus chanco* | Mongolia | Tsuda et al., 1997 | Mongolian wolf |
| Seq39 | KC414568.1 | *Canis lupus chanco* | Mongolia | Dou and Zhang, 2012 | Mongolian wolf |
| Seq40 | KC414567.1 | *Canis lupus chanco* | Mongolia | Dou and Zhang, 2012 | Mongolian wolf |
| Seq41 | KC414566.1 | *Canis lupus chanco* | Mongolia | Dou and Zhang, 2012 | Mongolian wolf |
| Seq42 | KC414565.1 | *Canis lupus chanco* | Mongolia | Dou and Zhang, 2012 | Mongolian wolf |
| Seq43 | KC414564.1 | *Canis lupus chanco* | Mongolia | Dou and Zhang, 2012 | Mongolian wolf |
| Seq44 | AB480744.1 | *Canis lupus chanco* | Koria | Ishiguro et al., 2009 | Japanese wolf |
| Seq45 | AB480743.1 | *Canis lupus chanco* | Japan | Ishiguro et al., 2009 | Japanese wolf |
| Seq46 | KT321361.1 | *Canis lupus familiaris* | Nepal | Chetri et al., 2016 |  |
| Seq47 | KT321361.1 | *Canis lupus chanco* | Nepal | Chetri et al., 2016 |  |
| Seq48 | EU442884.2 | *Canis lupus chanco* | Mongolia | Meng and Zhang, 2008 | Mongolian wolf |
| Seq49 | KC414576.1 | *Canis lupus chanco* | Mongolia | Dou and Zhang, 2012* | Mongolian wolf |
| Seq50 | KC414575.1 | *Canis lupus chanco* | Mongolia | Dou and Zhang, 2012 | Mongolian wolf |
| Seq51 | KC414574.1 | *Canis lupus chanco* | Mongolia | Dou and Zhang, 2012 | Mongolian wolf |
| Seq52 | KC414573.1 | *Canis lupus chanco* | Mongolia | Dou and Zhang, 2012 | Mongolian wolf |
| Seq53 | KC414572.1 | *Canis lupus chanco* | Mongolia | Dou and Zhang, 2012 | Mongolian wolf |
| Seq54 | KC414571.1 | *Canis lupus chanco* | Mongolia | Dou and Zhang, 2012 | Mongolian wolf |
| Seq55 | KC414570.1 | *Canis lupus chanco* | Mongolia | Dou and Zhang, 2012 | Mongolian wolf |
| Seq56 | KC414569.1 | *Canis lupus chanco* | Mongolia | Dou and Zhang, 2012 | Mongolian wolf |
| Seq57 | JX415353.1 | *Canis lupus chanco* | Mongolia | Dou, 2012* | Mongolian wolf |
| Seq58 | JX415352.1 | *Canis lupus chanco* | Mongolia | Dou, 2012 | Mongolian wolf |
| Seq59 | JX415351.1 | *Canis lupus chanco* | Mongolia | Dou, 2012 | Mongolian wolf |
| Seq60 | JX415350.1 | *Canis lupus chanco* | Mongolia | Dou, 2012 | Mongolian wolf |
| Seq61 | JX415349.1 | *Canis lupus chanco* | Mongolia | Dou, 2012 | Mongolian wolf |
| Seq62 | JX415348.1 | *Canis lupus chanco* | Mongolia | Dou, 2012 | Mongolian wolf |
| Seq63 | JX415347.1 | *Canis lupus chanco* | Mongolia | Dou, 2012 | Mongolian wolf |
| Seq64 | JX415346.1 | *Canis lupus chanco* | Mongolia | Dou, 2012 | Mongolian wolf |
| Seq65 | JX415345.1 | *Canis lupus chanco* | Mongolia | Dou, 2012 | Mongolian wolf |
| Seq66 | JX415343.1 | *Canis lupus chanco* | Mongolia | Dou, 2012 | Mongolian wolf |
| Seq67 | AY333741.1 | *Canis lupus chanco* | India | Sharma et al., 2003 |  |
| Seq68 | AY333740.1 | *Canis lupus chanco* | India | Sharma et al., 2003 |  |
| Seq69 | AY333739.1 | *Canis lupus chanco* | India | Sharma et al., 2003 |  |
| Seq70 | AY289994.1 | *Canis himalayensis* | India | Aggarwal et al., 2007 |  |
| Seq71 | AY289993.1 | *Canis himalayensis* | India | Aggarwal et al., 2007 |  |
| Seq72 | AY289992.1 | *Canis himalayensis* | India | Aggarwal et al., 2007 |  |
| Seq73 | AY289991.1 | *Canis himalayensis* | India | Aggarwal et al., 2007 |  |
| Seq74 | AY289990.1 | *Canis himalayensis* | India | Aggarwal et al., 2007 |  |
| Seq75 | AY289989.1 | *Canis himalayensis* | India | Aggarwal et al., 2007 |  |
| Seq76 | AY289988.1 | *Canis himalayensis* | India | Aggarwal et al., 2007 |  |
| Seq77 | AY289987.1 | *Canis himalayensis* | India | Aggarwal et al., 2007 |  |
| Seq78 | AY289986.1 | *Canis himalayensis* | India | Aggarwal et al., 2007 |  |
| Seq79 | AY289985.1 | *Canis himalayensis* | India | Aggarwal et al., 2007 |  |
| Seq80 | AY289983.1 | *Canis himalayensis* | India | Aggarwal et al., 2007 |  |
| Seq81 | AY289982.1 | *Canis himalayensis* | India | Aggarwal et al., 2007 |  |
| Seq82 | AY289981.1 | *Canis himalayensis* | India | Aggarwal et al., 2007 |  |
| Seq83 | AY289980.1 | *Canis himalayensis* | India | Aggarwal et al., 2007 |  |
| Seq84 | AY289979.1 | *Canis himalayensis* | India | Aggarwal et al., 2007 |  |
| Seq85 | AY289978.1 | *Canis himalayensis* | India | Aggarwal et al., 2007 |  |
| Seq86 | AY289977.1 | *Canis himalayensis* | India | Aggarwal et al., 2007 |  |
| Seq87 | 1643-SK-1 | *Canis lupus chanco* | India | Present Study |  |
| Seq88 | 5419-JK | *Canis lupus chanco* | India | Present Study |  |
| Seq89 | 5412-JK | *Canis lupus lupus* | India | Present Study |  |
| Seq90 | 5413-JK | *Canis lupus lupus* | India | Present Study |  |
| Seq91 | AB007372.1 | *Canis lupus lupus* | Mongolia | Tsuda et al., 1997 |  |
| Seq92 | AB007373 | *Canis lupus lupus* | Mongolia | Tsuda et al., 1998 |  |
| Seq93 | KX898331 | *Canis lupus lupus* | Greenland | Ersmark et al. 2016 |  |
| Seq94 | AF008141 | *Canis lupus lupus* | Saudi Arabia | Vila et al. 1997 |  |
| Seq95 | AF 098125 | *Canis lupus lupus* | Canada | Kopp et al. (unpublished) |  |
| Seq96 | DQ 480508 | *Canis lupus lupus* | Canada | Björnerfeldt et al. 2006 |  |
| Seq97 | FJ 978035 | *Canis lupus lupus* | Europe | Pilot et al. 2010* |  |
| Seq98 | AM 711902 | *Canis lupus lupus* | Sweden | Arnason et al. 2007 |  |
| Seq99 | FJ 978024 | *Canis lupus lupus* | Europe | Pilot et al. 2010 |  |
| Seq100 | KF 661058 | *Canis lupus lupus* | Alaska | Thalmann et al. 2013 |  |
| Seq101 | KF 661064 | *Canis lupus lupus* | United States of America | Thalmann et al. 2013 |  |

*Based on NCBI GenBank submission year and authors
