# Supplementary material for: Revisiting the Woolly wolf (Canis lupus chanco) phylogeny in Himalaya: Addressing taxonomy, spatial extent and distribution of an ancient lineage in Asia
Source: PLoS One. 2020 Apr 16;15(4):e0231621. doi: 10.1371/journal.pone.0231621 (PMC7162449; doi:10.1371/journal.pone.0231621)
Supplement: S2 Table — (DOCX) [file pone.0231621.s002.docx]

Table S2. Sequence divergence between the different haplotypes (H1-H15) of Woolly wolf (*Canis lupus chanco)* using a mitochondrial control region

|  | H1 | H2 | H3 | H4 | H5 | H6 | H7 | H8 | H9 | H10 | H11 | H12 | H13 | H14 | H15 |
| --- | --- | --- | --- | --- | --- | --- | --- | --- | --- | --- | --- | --- | --- | --- | --- |
| H1 |  |  |  |  |  |  |  |  |  |  |  |  |  |  |  |
| H2 | 0.014 |  |  |  |  |  |  |  |  |  |  |  |  |  |  |
| H3 | 0.014 | 0.009 |  |  |  |  |  |  |  |  |  |  |  |  |  |
| H4 | 0.014 | 0.000 | 0.009 |  |  |  |  |  |  |  |  |  |  |  |  |
| H5 | 0.023 | 0.009 | 0.018 | 0.009 |  |  |  |  |  |  |  |  |  |  |  |
| H6 | 0.019 | 0.005 | 0.014 | 0.005 | 0.014 |  |  |  |  |  |  |  |  |  |  |
| H7 | 0.019 | 0.014 | 0.014 | 0.014 | 0.023 | 0.019 |  |  |  |  |  |  |  |  |  |
| H8 | 0.009 | 0.005 | 0.005 | 0.005 | 0.014 | 0.009 | 0.009 |  |  |  |  |  |  |  |  |
| H9 | 0.034 | 0.034 | 0.034 | 0.034 | 0.034 | 0.039 | 0.039 | 0.029 |  |  |  |  |  |  |  |
| H10 | 0.005 | 0.009 | 0.009 | 0.009 | 0.018 | 0.014 | 0.014 | 0.005 | 0.029 |  |  |  |  |  |  |
| H11 | 0.009 | 0.023 | 0.023 | 0.023 | 0.033 | 0.028 | 0.028 | 0.019 | 0.044 | 0.014 |  |  |  |  |  |
| H12 | 0.019 | 0.014 | 0.005 | 0.014 | 0.023 | 0.019 | 0.019 | 0.009 | 0.039 | 0.014 | 0.028 |  |  |  |  |
| H13 | 0.014 | 0.009 | 0.009 | 0.009 | 0.019 | 0.014 | 0.014 | 0.005 | 0.034 | 0.009 | 0.023 | 0.014 |  |  |  |
| H14 | 0.009 | 0.005 | 0.005 | 0.005 | 0.014 | 0.009 | 0.009 | 0.001 | 0.029 | 0.005 | 0.019 | 0.009 | 0.005 |  |  |
| H15 | 0.019 | 0.023 | 0.023 | 0.023 | 0.033 | 0.028 | 0.028 | 0.019 | 0.044 | 0.014 | 0.009 | 0.028 | 0.024 | 0.019 |  |
