# Supplementary material for: Revisiting the Woolly wolf (Canis lupus chanco) phylogeny in Himalaya: Addressing taxonomy, spatial extent and distribution of an ancient lineage in Asia
Source: PLoS One. 2020 Apr 16;15(4):e0231621. doi: 10.1371/journal.pone.0231621 (PMC7162449; doi:10.1371/journal.pone.0231621)
Supplement: S3 Table — (DOCX) [file pone.0231621.s003.docx]

Table S3. Inter species genetic distance of the family canidae using control regions marker.

|  |  | 1 | 2 | 3 | 4 | 5 | 6 | 7 | 8 | 9 | 10 | 11 | 12 | 13 | 14 | 15 | 16 |
| --- | --- | --- | --- | --- | --- | --- | --- | --- | --- | --- | --- | --- | --- | --- | --- | --- | --- |
| 1 | *Canis lupus chanco (*Uttarakhand) |  |  |  |  |  |  |  |  |  |  |  |  |  |  |  |  |
| 2 | *Canis lupus indica* (India mainland) | 0.095 |  |  |  |  |  |  |  |  |  |  |  |  |  |  |  |
| 3 | *Canis lupus pallipes* (India mainland) | 0.095 | 0.000 |  |  |  |  |  |  |  |  |  |  |  |  |  |  |
| 4 | *Canis lupus chanco* (Nepal) | 0.007 | 0.086 | 0.086 |  |  |  |  |  |  |  |  |  |  |  |  |  |
| 5 | *Canis lupus lupus (*Jammu & Kashmir*)* | 0.096 | 0.062 | 0.062 | 0.104 |  |  |  |  |  |  |  |  |  |  |  |  |
| 6 | *Canis lupus lupus* | 0.086 | 0.086 | 0.086 | 0.094 | 0.053 |  |  |  |  |  |  |  |  |  |  |  |
| 7 | *Cuon alpinus* | 0.163 | 0.163 | 0.163 | 0.173 | 0.155 | 0.107 |  |  |  |  |  |  |  |  |  |  |
| 8 | *Canis lupus* (Greenland) | 0.069 | 0.069 | 0.069 | 0.077 | 0.038 | 0.015 | 0.125 |  |  |  |  |  |  |  |  |  |
| 9 | *Canis lupus* (Saudi Arebia) | 0.096 | 0.062 | 0.062 | 0.104 | 0.000 | 0.053 | 0.155 | 0.038 |  |  |  |  |  |  |  |  |
| 10 | *Canis lupus* (Canada) | 0.069 | 0.069 | 0.069 | 0.077 | 0.038 | 0.015 | 0.125 | 0.000 | 0.038 |  |  |  |  |  |  |  |
| 11 | *Canis lupus* (Canada) | 0.069 | 0.069 | 0.069 | 0.077 | 0.038 | 0.015 | 0.125 | 0.000 | 0.038 | 0.000 |  |  |  |  |  |  |
| 12 | *Canis lupus lupus* (Sweden) | 0.094 | 0.094 | 0.094 | 0.102 | 0.061 | 0.007 | 0.115 | 0.022 | 0.061 | 0.022 | 0.022 |  |  |  |  |  |
| 13 | *Canis lupus* (Alaska) | 0.086 | 0.086 | 0.086 | 0.094 | 0.046 | 0.045 | 0.163 | 0.030 | 0.046 | 0.030 | 0.030 | 0.053 |  |  |  |  |
| 14 | *Canis lupus* (USA) | 0.078 | 0.077 | 0.077 | 0.086 | 0.045 | 0.037 | 0.152 | 0.037 | 0.045 | 0.037 | 0.037 | 0.029 | 0.053 |  |  |  |
| 15 | *Chrysocyon brachyurus* | 0.134 | 0.162 | 0.162 | 0.143 | 0.143 | 0.152 | 0.161 | 0.153 | 0.143 | 0.153 | 0.153 | 0.162 | 0.143 | 0.143 |  |  |
| 16 | *Canis aureus* | 0.159 | 0.141 | 0.141 | 0.150 | 0.181 | 0.180 | 0.217 | 0.170 | 0.181 | 0.170 | 0.170 | 0.189 | 0.169 | 0.198 | 0.193 |  |
