# Supplementary material for: Revisiting the Woolly wolf (Canis lupus chanco) phylogeny in Himalaya: Addressing taxonomy, spatial extent and distribution of an ancient lineage in Asia
Source: PLoS One. 2020 Apr 16;15(4):e0231621. doi: 10.1371/journal.pone.0231621 (PMC7162449; doi:10.1371/journal.pone.0231621)
Supplement: S4 Table — (DOCX) [file pone.0231621.s004.docx]

Table S4. AMOVA results of Woolly wolf in phylogenetic tree of different clades.

| Source of variation | df | Sum of squares | Variance Components | Percentage of Variation |
| --- | --- | --- | --- | --- |
| AMongoliagolia populations | 6 | 58.421 | 1.36698 Va | 71.35 |
| Within populations | 54 | 29.644 | 0.54897 Vb | 28.65 |
| Total | 60 | 88.066 | 1.91595 | 100.0 |
| Fixation Index F*_ST_*: 0.71347; P=0.000 | | | |  |
