# Supplementary material for: Revisiting the Woolly wolf (Canis lupus chanco) phylogeny in Himalaya: Addressing taxonomy, spatial extent and distribution of an ancient lineage in Asia
Source: PLoS One. 2020 Apr 16;15(4):e0231621. doi: 10.1371/journal.pone.0231621 (PMC7162449; doi:10.1371/journal.pone.0231621)
Supplement: S1 Fig — (DOCX) [file pone.0231621.s005.docx]

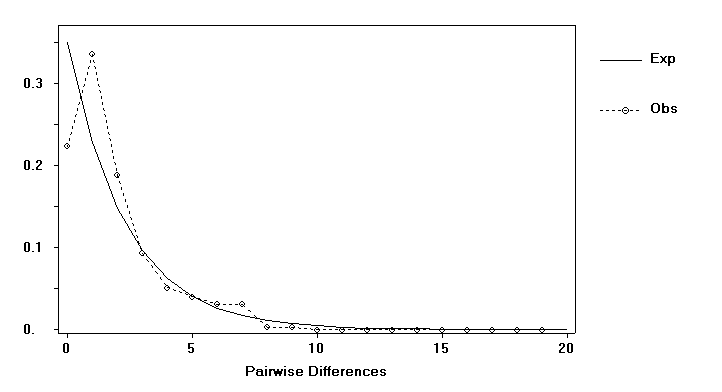


Figure S1. Pairwise mismatch distribution graph of *Canis lupus chanco* using a mitochondrial control region.
