# Supplementary material for: Revisiting the Woolly wolf (Canis lupus chanco) phylogeny in Himalaya: Addressing taxonomy, spatial extent and distribution of an ancient lineage in Asia
Source: PLoS One. 2020 Apr 16;15(4):e0231621. doi: 10.1371/journal.pone.0231621 (PMC7162449; doi:10.1371/journal.pone.0231621)
Supplement: S2 Fig — Map showing the clade wise distribution of samples in their distribution range. Values above the nodes are posterior probabilities. (DOCX) [file pone.0231621.s006.docx]

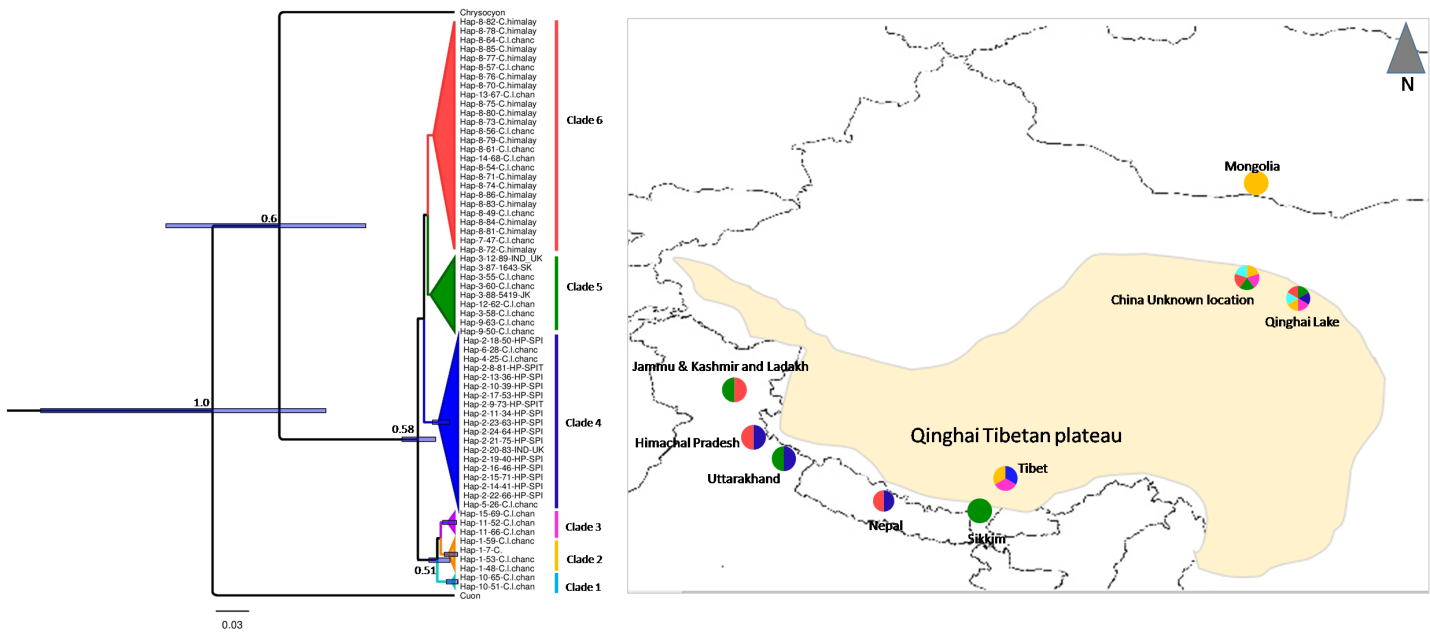


Figure S2. Phylogenetic analysis based on Bayesian inferences (BI) tree constructed of *Canis lupus chanco* using mitochondrial control region of *Cuon alpines* and *Chrysocyon brachyurus* as the out group. Map showing the clade wise distribution of samples in their distribution range. Values above the nodes are posterior probabilities
